# High Conduction Band Inorganic Layers for Distinct Enhancement of Electrical Energy Storage in Polymer Nanocomposites

**DOI:** 10.1007/s40820-022-00902-9

**Published:** 2022-07-25

**Authors:** Yingke Zhu, Zhonghui Shen, Yong Li, Bin Chai, Jie Chen, Pingkai Jiang, Xingyi Huang

**Affiliations:** 1 Shanghai Key Laboratory of Electrical Insulation and Thermal Ageing, State Key Laboratory of Metal Matrix Composites, Department of Polymer Science and Engineering, Shanghai Jiao Tong University, Shanghai, 200240 People’s Republic of China; 2 State Key Laboratory of Advanced Technology for Materials Synthesis and Processing, Center of Smart Materials and Devices, Wuhan University of Technology, Wuhan, 430070 People’s Republic of China; 3 Institute of Applied and Physical Chemistry and Center for Environmental Research and Sustainable Technology, University of Bremen, 28359 Bremen, Germany

**Keywords:** Boron nitride nanosheet, Conduction band, Efficiency, Energy density, Barrier

## Abstract

Dielectric polymer nanocomposites are considered as one of the most promising candidates for high-power-density electrical energy storage applications. Inorganic nanofillers with high insulation property are frequently introduced into fluoropolymer to improve its breakdown strength and energy storage capability. Normally, inorganic nanofillers are thought to introducing traps into polymer matrix to suppress leakage current. However, how these nanofillers effect the leakage current is still unclear. Meanwhile, high dopant (> 5 vol%) is prerequisite for distinctly improved energy storage performance, which severely deteriorates the processing and mechanical property of polymer nanocomposites, hence brings high technical complication and cost. Herein, boron nitride nanosheet (BNNS) layers are utilized for substantially improving the electrical energy storage capability of polyvinylidene fluoride (PVDF) nanocomposite. Results reveal that the high conduction band minimum of BNNS produces energy barrier at the interface of adjacent layers, preventing the electron in PVDF from passing through inorganic layers, leading to suppressed leakage current and superior breakdown strength. Accompanied by improved Young’s modulus (from 1.2 GPa of PVDF to 1.6 GPa of nanocomposite), significantly boosted discharged energy density (14.3 J cm^−3^) and charge–discharge efficiency (75%) are realized in multilayered nanocomposites, which are 340 and 300% of PVDF (4.2 J cm^−3^, 25%). More importantly, thus remarkably boosted energy storage performance is accomplished by marginal BNNS. This work offers a new paradigm for developing dielectric nanocomposites with advanced energy storage performance.

**Supplementary Information:**

The online version contains supplementary material available at 10.1007/s40820-022-00902-9.

## Highlights

- High conduction band inorganic layers are manufactured via simple but efficient methodology.
- The multilayered nanocomposite possesses an outstanding breakdown strength of 611 MV m^−1^ and an excellent discharged energy density of 14.3 J cm^−3^, which are 119% and 177% of the randomly dispersed nanocomposite (515 MV m^−1^, and 8.1 J cm^−3^).
- The current work offers a new paradigm for design and production of high energy density flexible dielectric films.

**Supplementary Information:**

The online version contains supplementary material available at 10.1007/s40820-022-00902-9.

## Graphical abstract

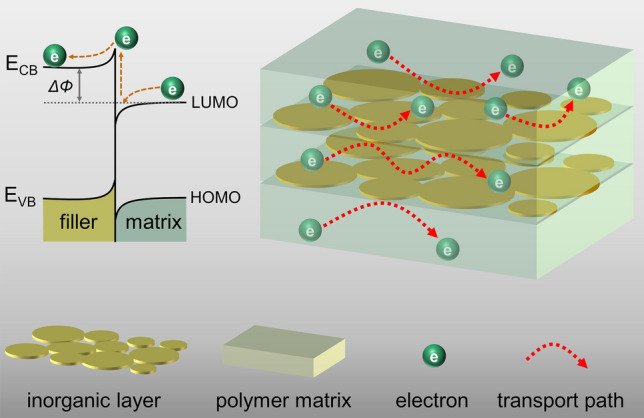

## Introduction

Due to rapid development of modern industry, stringent requirements are put forward for electronic systems, including compactness, flexibility, good reliability under harsh environment [1–3]. Dielectric capacitors are major components in modern electronics due to its highest power density (benefiting from its highest discharge rate) in comparison to other energy storage counterparts [4–10], which can be applied to electric pulse systems, defibrillators, hybrid vehicles, oil and gas exploration. However, the energy density of polymer dielectrics is lower than other energy storage materials, such as batteries, supercapacitors. For example, the energy density of commercialized biaxially oriented polyproplene (BOPP) is lower than 4 J cm^−3^ [11–13], which causes its cumbersome volume and weight in practical application.

Generally, the energy density (usually referring to discharged energy density, *U*_*e*_) could be derived from ${U}_{e}=\int EdD$, where *E* is applied electric field and *D* is the electric displacement under *E*. For linear dielectrics, such as BOPP, *U*_*e*_ is determined by ${U}_{e}=1/2{\varepsilon }_{0}{\varepsilon }_{r}{E}_{b}^{2}$, where *ε*_*0*_ denotes the vacuum permittivity, *ε*_*r*_ is the dielectric constant, and *E*_*b*_ is the breakdown strength. Apparently, the *U*_*e*_ of dielectric is determined by its *ε*_*r*_ and *E*_*b*_. To date, two strategies seem to be effective in enhancing the *U*_*e*_ of polymer composites. One is utilizing high-k nanofiller to realize a concurrent increase of *ε*_*r*_ and *E*_*b*_, which can be divided into two aspects, synthesizing hybrid high-k nanofiller and designing the hierarchical structure of dielectric films [14–16]. However, these two approaches both are tanglesome (either in nanofiller synthesis or film fabricating process). The other is utilizing wide bandgap nanofiller (i.e., boron nitride nanosheets (BNNSs) or Al_2_O_3_) to suppress the leakage current and hence enhance the *E*_*b*_ of polymer composites [17–19]. Nevertheless, a high content (normally exceeds 5 vol%) is prerequisite to ensure a great improvement of *E*_*b*_ [20–22]. Such high dopant not only damages the processibility and mechanical properties of composites, but also induces high cost because high quality BNNSs need tedious exfoliation process and plenty of time [16]. Therefore, substantially improvement of energy storage performance of dielectric nanocomposite with ultra-low dopant remains a challenge.

**Fig. 1:**
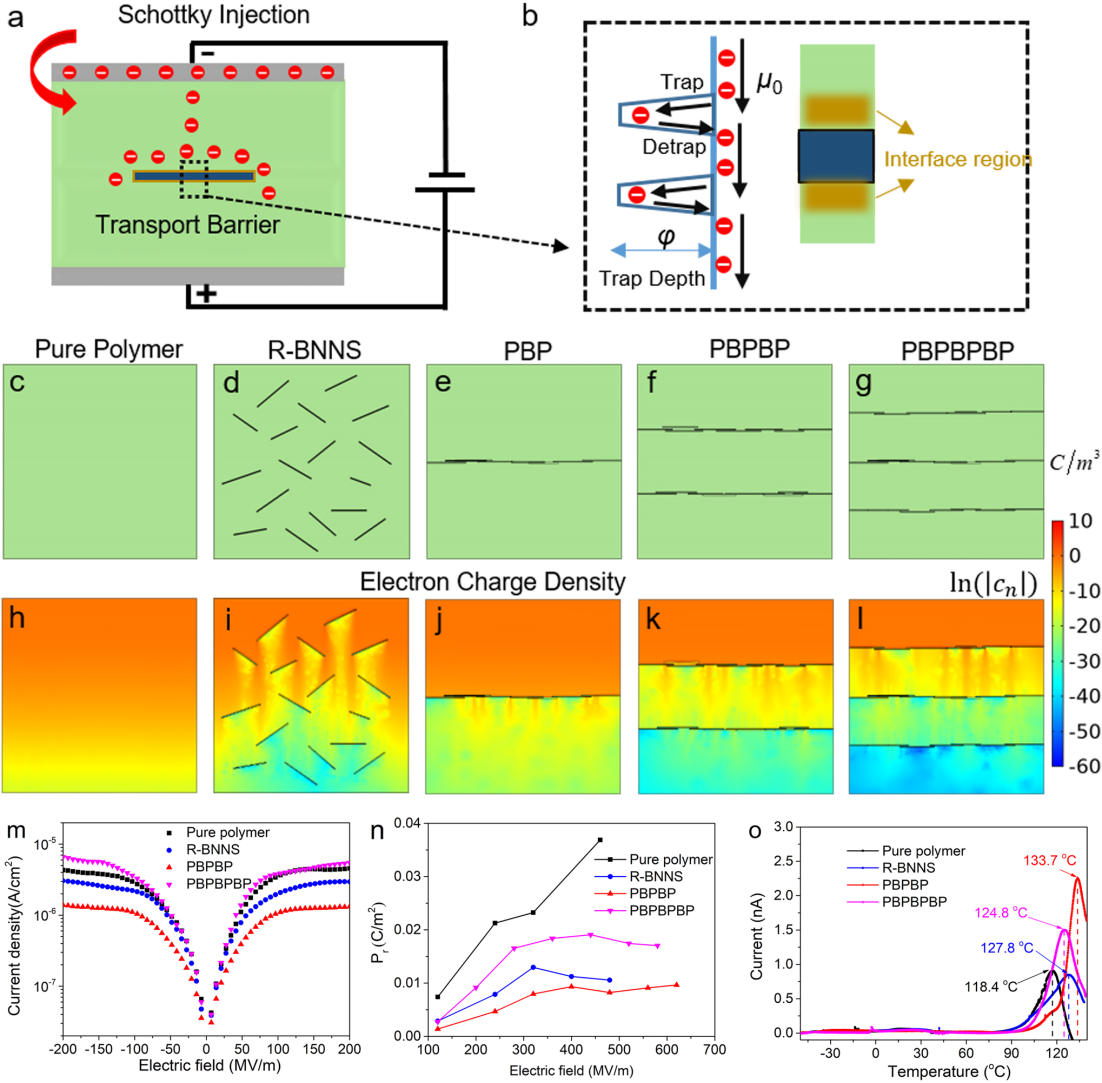
Schematic illustration of **a** Schottky injection in BNNS polymer composite and **b** electron transportation at the filler/matrix interface with trapping and detrapping process, **c** pure polymer, **d** nanocomposite with randomly dispersed BNNS (R-BNNS), **e** nanocomposite with one BNNS layer (PBP), **f** nanocomposite with two BNNS layers (PBPBP), **g** nanocomposite with three BNNS layers (PBPBPBP). Simulation results of the electron charge density distributions (*t* = 1 s) of **h** pure polymer, **i** nanocomposite with randomly dispersed BNNS, **j** nanocomposite with one BNNS layer, **k** nanocomposite with two BNNS layers, **l** nanocomposite with three BNNS layers, under the DC electric field of 300 MV m^−1^ and the temperature of 298 K. Comparison of **m** current density, **n** remnant polarization, and **o** TSDC results of PVDF, nanocomposite with randomly dispersed BNNS (R-BNNS), nanocomposite with two BNNS layers (PBPBP) and nanocomposite with three BNNS layers (PBPBPBP) in experiments

BNNS has received tremendous attention for suppressing the leakage current density and improving the breakdown strength of dielectric nanocomposites due to its wide bandgap (~ 6 eV) and excellent electrical insulation property [18, 22, 23]. However, how BNNS inhibits the leakage current density of dielectric nanocomposites is still unclear. Herein, in order to reduce the dopant content and uncover the intrinsic mechanism of suppressed leakage current, we propose the manufacture of multilayered nanocomposites followed by identifying the electronic structure of BNNS and polymer matrix. Firstly, we conduct computational simulation and experimental research to study the macroscopic charge behavior of nanocomposites with aligned BNNS layers and randomly dispersed BNNSs (Fig. 1). Results reveal that nanocomposite with layered BNNSs exhibits remarkably suppressed electron charge density when compared to the nanocomposite with randomly dispersed BNNSs, which is also confirmed in experimental results. In addition, layered BNNSs induces lower remnant polarization and deeper traps in nanocomposites than randomly dispersed BNNSs. Afterwards, the effect of BNNS layer on the mechanical, dielectric and energy storage properties of polyvinylidene fluoride (PVDF) nanocomposites are systematically studied. Different from traditional nanocomposites with nanofiller uniformly dispersed in polymer matrix [24–29], BNNSs is directly aligned and connected in present nanocomposites, giving rise to trace content but remarkably improved energy storage performance. For instance, polymer nanocomposites with 1 and 1.35 vol% possess the highest discharged energy density and highest charge–discharge efficiency of 14.3 J cm^−3^ and 75%, respectively, which is 340 and 300% of PVDF (4.2 J cm^−3^ and 25%), separately. Significantly, the electrons in PVDF cannot migrates through BNNS layer due to an energy barrier induced by high conduction band minimum of BNNS and relatively low lowest unoccupied molecular orbital (LUMO) of PVDF, which is direct evidence of how BNNSs suppress the leakage current density in dielectric nanocomposites. This work provides a new paradigm for designing dielectric nanocomposites with excellent energy storage capability .

## Experimental Section

### Materials

h-BN (003) was bought from 3 M Technical Ceramics (U.S.A.). PVDF was kindly provided by SOLVAY Group. DMF (dimethylformamide) was bought from Sinopharm Chemical Reagent Co., Ltd. Isopropanol was bought from Tansoole (China). All the reagents are AR grade.

### Exfoliation of h-BN to Boron Nitride Nanosheets

The exfoliation process was according to a previous process [30]. Briefly, 3 g of h-BN powder was dispersed into a 200 mL hybrid solution (isopropanol: deionized water = 1:1). Then, this solution was parked in an ultra-sonication machine for 4 h. Then, the obtained white mixture was centrifuged at 4000 rpm for 20 min to remove the thick h-BN and the supernatants were decanted. Afterwards, BNNSs were gathered by filtering the supernatants and during at vacuum oven at 70 °C for 12 h.

### Fabricating Process of Multilayered Nanocomposites and BNNS Random Dispersed Film

**Fig. 2:**
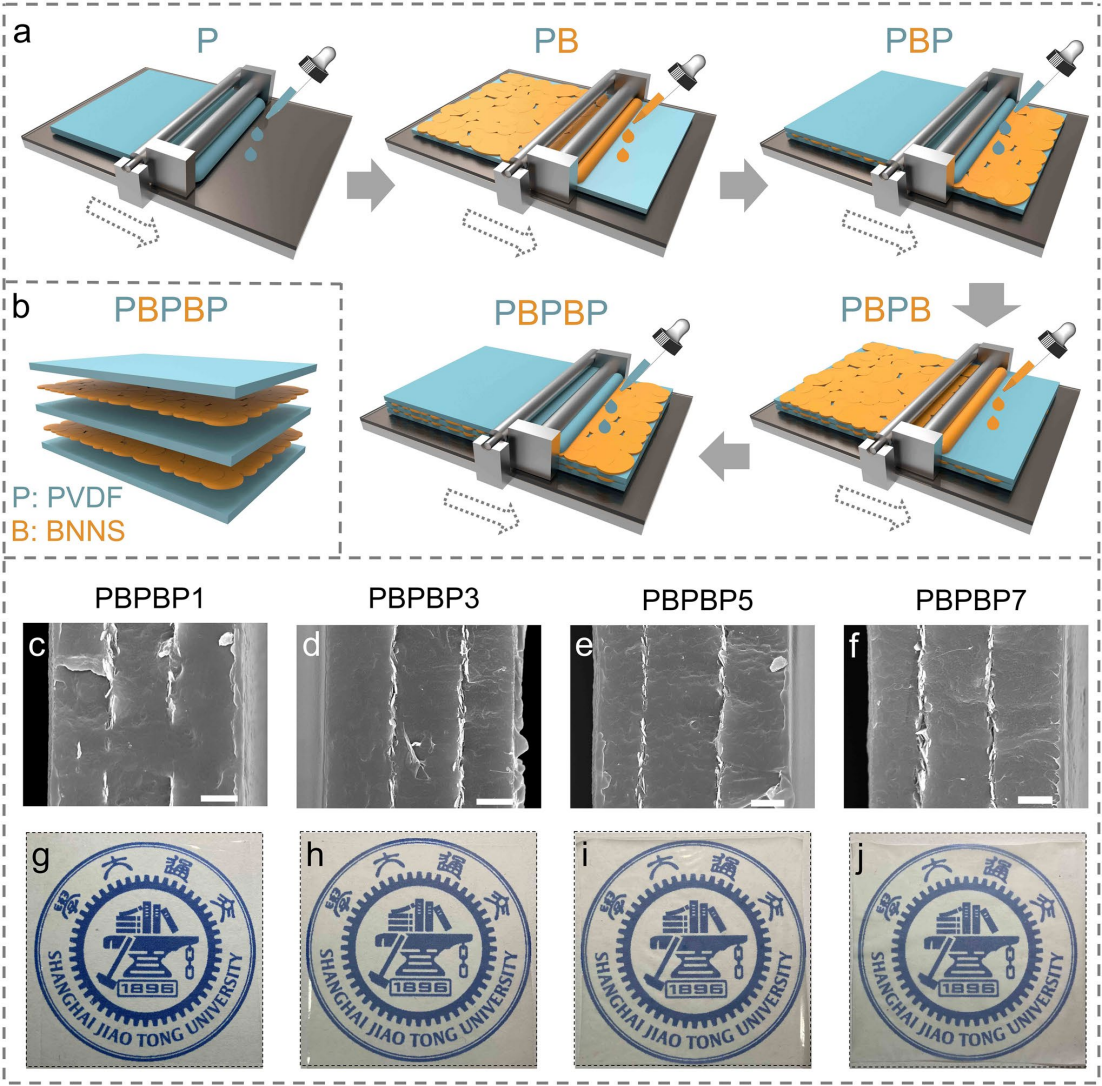
**a** Illustration of fabrication process of PBPBP films (P represents the PVDF layer and B is the BNNS layer). **b** Schematic illustration of PBPBP structure. Cross-sectional SEM images of **c** PBPBP1, **d** PBPBP3, **e** PBPBP5, and **f** PBPBP7 (the scale bar is 2 μm). Optical pictures of **g** PBPBP1, **h** PBPBP3, **i** PBPBP5, and **j** PBPBP7

The typical fabrication process of five layer structure films (hereafter termed as PBPBPs, s is the concentration of BNNSs solution, P and B represent PVDF and BNNS, respectively) was exhibited in Fig. 2a (later). PVDF/DMF solution was fabricated under vigorous stirring until the color turned into clear. Then, the first PVDF layer was fabricated by casting this solution onto a glass plate. Afterwards, the glass plate was dried in vacuum oven overnight. In addition, BNNSs were dispersed into isopropanol by an ultrasonic process to obtain a uniform solution with four weight concentrations (1, 3, 5, and 7 mg mL^−1^). Then, BNNSs solution was casted onto the PVDF layer, which is served as second layer. Next, the glass plate was placed in vacuum oven to remove the isopropanol. Then, three layers (PVDF, BNNSs, and PVDF) were casted onto BNNS layer one by one utilizing the same casting process. After dried in vacuum at 50 °C for 12 h to remove the trace solvent, these glass plates were placed at 200 °C for 7 min and immediately putted into ice water. Then, these PBPBP films were taken off from the substrates and dried overnight. These PBPBP films were denoted as PBPBP1, PBPBP3, PBPBP5, and PBPBP7, respectively (depending on the weight content of BNNSs in solution). PBPBPBP film was fabricated by using the same procedure. For preparation of BNNS random dispersed film, certain amount of BNNS were added into DMF solution with sonication for 1 h. Then, PVDF powder were added to the mixture with vigorous agitation for 24 h. Then, the mixture solution were casted onto a glass plate and put in vacuum at 50 °C for 12 h to remove trace DMF. The post-treatment process is the same with PBPBP films. For the sake of convenience, 1.3 wt% BNNSs random dispersed nanocomposite is named as R-BNNS.

### Characterization

The morphologies of nanocomposite filmsand BNNSs were studied by field emission scanning electron microscope (FE-SEM, Nova NanoSEM 450, FEI, USA) and transmission electron microscope (TEM, JEM-2100, JEOL Ltd., Japan). X-ray diffraction (XRD, D/max-2200/PC, Cu Kα source, Rigaku, Japan) and Fourier transform infrared spectroscopy (FT-IR, PerkinElmer Paragon 1000, 4000 to 400 cm^−1^) were managed to study the crystal form of samples. In order to detect the crystal and melting properties of samples, differential scanning calorimetry (DSC, NETZSCH 200 F3, Germany) was performed under N_2_ atmosphere at 10 °C min^−1^. In order to proof the weight percent of BNNSs in PBPBP films, thermal gravimetric analysis (TGA, NETZSCH TG209 F3, Germany) was conducted from 50 to 800 °C at a rate of 20 °C min^−1^. The volume content of BNNSs in composites were calculated by Eq. (1):

1 $$V_{B} = m_{B} \rho_{P} /\left( {m_{B} \rho_{P} + m_{P} \rho_{B} } \right) \times 100\%$$

where *m* and *ρ* denote the mass content and density of BNNS and PVDF, respectively. Subscript *B* and *P* represent BNNS and PVDF, respectively. In order to measure the dielectric and energy storage properties of samples, two sides of dielectric films were sputtered by copper served as electrodes with a diameter of 12 and 3 mm, respectively. Then, dielectric properties of dielectric films was studied by a Novocontrol Alpha-N dielectric analyzer (GmbH Concept 40) ranging from 0.1 Hz to 100 MHz under ambient and various temperature (− 50 to 100 °C). For energy storage measurement, precision multiferroic analyzer (Radiant technologies) were conducted to test the Electric displacement-electric field (D-E) loops (at 10 Hz) and leakage current densities of samples. In order to study the breakdown strength of as-prepared samples, a DC high voltage generator (Shanghai Juter High Voltage Electrical & Equipment Co. Ltd) was used with a ramping rate of 200 V s^−1^. In addition, to ensure the reliability of breakdown strength, each sample was tested with 15 different plots. In order to measure the Young’s modulus of samples, the stain-stress curves were conducted by INSTRON 3343 with a capacity of 1 kN under an extension rate of 8 mm min^−1^. Thermally stimulated depolarization current (TSDC) was conducted by using Keithley 6514 electrometer as follows. First, two sides of films were sputtered by copper served as electrodes with a diameter of 5 mm. Afterwards, the films were polarized under 30 MV m^−1^ at 50 °C for 10 min and then rapidly cooled to − 50 °C with keeping the applied electric field. Afterwards, the electric field were removed and then the samples were short circuited for 5 min. Finally, the films were heated to 140 °C with the heating rate of 3 °C min^−1^. Keithley 6514 electrometer was utlized to measure the current during heating. The cyclic charge–discharge measurement were conducted using PK-CPR1502 (Poly-K Technologies) under an electric field of 200 MV m^−1^.

### Simulation Section

Based on the Schottky mechanism, a unipolar electron injection and transport model is built to study the charge transport behavior in different microstructures. For simplification, the contribution of holes or ions and the process of recombination and other effects are not be considered in this work. Here, an apparent electron mobility *μ*_a_ is introduced to describe the trapping and detrapping process at the interface regions by Eq. (2)

2 $$\mu_{{\text{a}}} { = }\mu_{{0}} \exp \left( {{{ - \xi } \mathord{\left/ {\vphantom {{ - \xi } {k_{{\text{B}}} T}}} \right. \kern-\nulldelimiterspace} {k_{{\text{B}}} T}}} \right)$$

where *k*_B_ and *T* are the Boltzmann constant and temperature, *μ*_0_ and *ξ* are intrinsic electron mobility and trap depth (eV), respectively. In this work, *ξ* is set with a constant value of 0.12 eV and temperature *T* = 298 K. The current density *J*_c_ at the cathode is expressed by Eq. (3)

3 $$J_{{\text{c}}} = {\text{A}}T^{2} \exp \left( {\frac{{ - w_{i} }}{{k_{B} T}}} \right)\exp \left( {\frac{q}{{k_{B} T}}\sqrt {\frac{eE}{{4\pi \varepsilon }}} } \right)$$

where A is the Richardson constant, *w*_i_ is the Schottky barrier, *q* is elementary electron charge, *E* is the electric field and *ε* is the dielectric constant. Then, the charge behavior in dielectrics is governed by Poisson’s equation (Eq. (4)), transport equation (Eq. (5)) and continuity equation (Eq. (6)), as follows:

4 $$J\left( {{\mathbf{r}},t} \right) = \mu_{a} \left( {{\mathbf{r}},t} \right)n\left( {{\mathbf{r}},t} \right)E\left( {{\mathbf{r}},t} \right){ + }D\left( {{\mathbf{r}},t} \right)\nabla n\left( {{\mathbf{r}},t} \right)$$

5 $$\nabla^{{2}} \varphi \left( {{\mathbf{r}},t} \right) = - \frac{{n\left( {{\mathbf{r}},t} \right)}}{{\varepsilon_{{0}} \varepsilon_{{\text{r}}} \left( {{\mathbf{r}},t} \right)}}$$

6 $$\nabla J\left( {{\mathbf{r}},t} \right) + \frac{{\partial n\left( {{\mathbf{r}},t} \right)}}{\partial t} = s$$

where *n* is the charge density, *D* is the charge diffusion coefficient which could be calculated from *μ*_0_ by the Einstein relation ${D \mathord{\left/ {\vphantom {D \mu }} \right. \kern-\nulldelimiterspace} \mu } = {{k_{{\text{B}}} T} \mathord{\left/ {\vphantom {{k_{{\text{B}}} T} q}} \right. \kern-\nulldelimiterspace} q}$, *φ* is the electric potential and s is source term. In order to describe different microstructures, a field variable $\eta$ is used to define different phases in composites by assigning various values, and then all position-dependent parameters will be determined. The parameters used in this simulation is presented in Table S1.

The first-principles calculations were performed in the frame of density functional theory (DFT) with the Vienna ab initio simulation package (VASP). The exchange–correlation energy is described by the Perdew-Burke-Ernzerhof (PBE) form of generalized-gradient approximation (GGA) exchange–correlation energy functional. The structure optimizations of h-BN and alpha-PVDF have been carried out by allowing all atomic positions to vary and fixing lattice parameters until the energy difference of successive atom configurations was less than 10^–4^ eV. The force on each atom in the relaxed structures was less than 0.015 eV Å^−1^. The cutoff energy for the plane-wave basis set was set to 400 eV. The k-point spacing was set to be smaller than 0.03 Å^−1^ over Brillouin zone (BZ). All computational models of PVDF and BNNS were performed using spin-polarized DFT as implemented in the CP2K quantum-chemical package. The electronic property of BNNS and PVDF is listed in Table S2.

## Results and Discussion

### Theoretical and Experimental Study of Nanocomposites with Different BNNSs Spatial Arrangement

BNNSs have shown remarkable ability of suppressing current density and improving breakdown strength of dielectric nanocomposites because its wide bandgap and two-dimensional features [31, 32]. However, in previous experiments, limited by the fabrication technology, BNNSs are usually randomly distributed in composites, which may weaken their two-dimensional shape effect on substantial improvement of dielectric properties [33–35]. In order to find solutions to maximize the insulating features of BNNSs, we take a unipolar charge injection and transport model as the example to study the microstructure effect on the ability of charge transport and then give some theoretically guidance on regulation of composite microstructures, as illustrated in Fig. 1a. In dielectric composites, the traps at the interface may influence the charge transport process in composites and thus affect the macroscopic dielectric performance, as sketched in Fig. 1b [36, 37]. Therefore, an apparent electron mobility is introduced to describe the trapping and detrapping process in this model, and more details are provided in simulation section.

Here, five representative microstructures are considered: pure polymer, traditional BNNSs random dispersed nanocomposite (R-BNNS), composite with one aligned BNNS layers (PBP), composite with two aligned BNNS layers (PBPBP), and composite with three aligned BNNS layers (PBPBPBP), as shown in Fig. 1c-g. As the simulation results shown in Fig. 1h-i, R-BNNS exhibits lower electron charge density than pure polymer because of high insulating BNNSs block the electron charge transport. As shown in Fig. 1j-l, when BNNSs are assembled into a compact and oriented layer, the blocking effect could be greatly enhanced, leading to an ultra-low electron density. Therefore, the orientation and distribution of BNNSs is closely related with the charge transport and corresponding dielectric properties. Meanwhile, one can see that PBPBP possesses lower electron charge density than PBP. After adding another BNNS layer, the electron charge density of PBPBPBP only displays a slightly decrease compared to PBPBP. Therefore, nanocomposite with two BNNS layers seems to possess lowest leakage current density when compared to other four dielectrics.

Afterwards, based on the simulation results, pure PVDF, nanocomposite with BNNS random dispersed, nanocomposite with two aligned BNNS layers (PBPBP), and nanocomposite with three aligned BNNS layers (PBPBPBP) are fabricated. The microstructure of the three nanocomposites are shown in Fig. S1, indicating the successful fabrication of dielectric films without defects. First, the current density and remnant polarization (*P*_r_), which are closely related to the charge density in dielectrics, of three nanocomposites and pure PVDF are explored. As shown in Fig. 1m, n, nanocomposite with two BNNS layers exhibits the lowest current density and *P*_r_ in four samples, revealing good consistency with simulation results. Afterwards, the thermally stimulated depolarization current (TSDC) are conducted to investigate the trap depth of three samples and results are shown in Fig. 1o. Clearly, the peak temperature of PBPBP (133.7 °C) is higher than pure polymer (118.4 °C), R-BNNS (127.8 °C) and PBPBPBP (124.8 °C), indicating that PBPBP could generate depper traps compared to pure polymer, R-BNNS and PBPBPBP [38]. This might be attributed to the successive and aligned BNNS layer, which is perpendicular to the direction of electric field, giving rise to deeper trap depth than randomly dispersed BNNSs.

Therefore, combining the simulation and experimental results, PBPBP seems to exhibting superior energy storage performance than pure polymer, R-BNNS, PBP and PBPBPBP. Furthermore, the breakdown strength and energy storage properties of R-BNNS and PBPBP are also compared in Fig. S3. Benefiting from suppressed current density, PBPBP exhibits much higher breakdown strength of 611 MV m^−1^, which is 120% of R-BNNS. In addition, PBPBP can bear an electric field of 620 MV m^−1^ with an efficiency of 72.3%, giving rise to a superior discharged energy density of 14.3 J cm^−3^, 1.77 times of R-BNNS (8.1 J cm^−3^).

### Preparation of PBPBP Films

Based on the experimental and simulation results, a series of PBPBP films were fabricated. To ensure high breakdown strength and hence superior energy storage performance of dielectric films, an exfoliation process of h-BN to BNNSs was conducted according to our previous work [30, 39, 40]. As shown in Fig. S2, after an ultra-sonication process of 4 h and centrifugation process (4000 rpm, 20 min), few-layered BNNSs were successfully acquired. The typical fabrication process of five layer structure films (hereafter termed as PBPBPs, s is the concentration of BNNSs solution) is shown in Fig. 2a. Briefly, this process includes five individual solution-casting moves, in which PVDF layer and BNNS layer are alternately arranged. The latter layer is casted after the previous layer is fully dried (the detailed process is shown in experimental section). To ensure a better illustration of thus prepared films, Fig. 2b exhibits the typical structure of PBPBP films. Figure 2c-f exhibit the cross-sectional SEM images of PBPBP1, PBPBP3, PBPBP5, and PBPBP7, respectively. Clearly, the BNNS layer in four films all processes a parallel direction along with the film. In addition, the continuity of BNNS layer grows better with increasing BNNSs content. Meanwhile, the thickness of BNNS layer exhibits a gradual increase from PBPBP1 to PBPBP7. As evidenced in Fig. S4c, the contents of BNNSs are 0.56, 1.00, 1.35, and 1.96 vol% for PBPBP1, PBPBP3, PBPBP5, and PBPBP7, respectively. In addition, all PBPBP films reveal a good transparency due to the ultrathin property of BNNSs.

To better understanding the effect of BNNS layer on the dielectric and energy storage performances of composite films, FT-IR and XRD are conducted and shown in Fig. S4a and b, respectively. The peaks at 976, 765, and 614 cm^−1^ of FT-IR and 18.6, 20.2° of XRD indicate that all dielectric films exhibiting *α* form [41, 42]. In addition, DSC is conducted to evaluate the crystallization behaviors of as-prepared dielectric films. As shown in Fig. S4d and f, the crystallinity of PBPBP films exhibits a minor increase (39.1 to 40.9%) comparing with PVDF (36.5%). Furthermore, as shown in Fig. S4e, comparing with pristine PVDF (140 °C), the crystallization temperature of PBPBP films show a gradual increase (up to 146 °C). These results indicate that BNNSs may act as nucleating agent for PVDF and increase the crystallinity of PVDF.

### Dielectric Properties of PBPBP Films under Ambient and Various Temperature

**Fig. 3:**
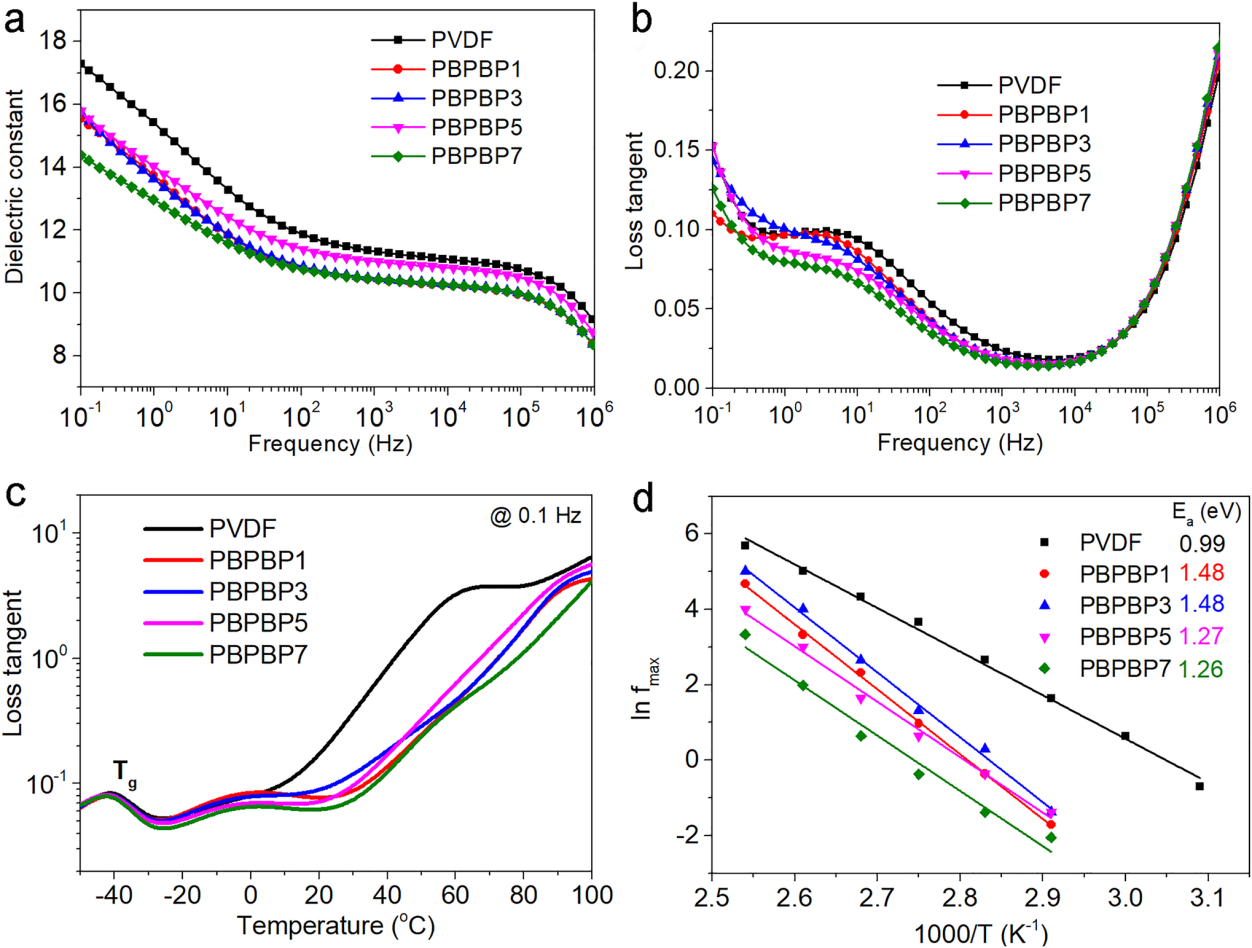
Frequency dependency of **a** dielectric constant and **b** loss tangent (tan*δ*) of PVDF and PBPBP films. Comparison of **c** loss tangent (tan*δ*) at 0.1 Hz under various temperature and **d** activation energy derived from Arrhenius plots (ln *f*_max_ vs. 1/*T*) of PVDF and PBPBP films

In order to study the effect of two BNNS layers on the dielectric properties of PBPBP films, broadband dielectric spectroscopy measurement is conducted and results are shown in Fig. 3. The dielectric constant of PBPBP films exhibit a gradually decrease comparing with pristine PVDF. For example, at 100 Hz, the dielectric constant of PBPBP7 and PVDF are 10.7 and 11.8, respectively. This is mainly roots in the low dielectric constant of BNNSs (~ 3.9) [43], which is nearly one third of pristine PVDF. More importantly, as shown in Fig. 3b, PBPBP films exhibit a significantly suppressed dielectric loss, especially at low frequencies (10^–1^ ~ 10^3^ Hz). For instance, at 10 Hz, the dielectric loss of PBPBP films reach as low as 0.067 for PBPBP7, exhibiting a 29% decrease comparing with pristine PVDF (0.094). This indicates that two BNNS layers can effectively suppress the dielectric loss derived from ion conduction [44–47].

To further evaluate the effect of BNNS layers on the dielectric properties of PBPBP films, dielectric performances of as-prepared composite films under various temperature are conducted. Figure S5 display the imaginary part of dielectric constant (*ε*_r_") of as-prepared samples. An upturn for *ε*_r_" at low frequencies around − 40 °C can be clearly seen for all five samples, which represents the glass transition temperature (*T*_g_) of PVDF. In addition, this upturn temperature increases with increasing frequency, indicating that the molecular relaxation requires more energy to occur under high frequencies. Furthermore, as shown in Fig. 3c, the conduction loss originated from impurity ions of PVDF, at temperature over 10 °C, is notably suppressed by BNNS layers [48].

Since the interfacial property of dielectric composite largely determines its energy storage performance, 3D mapping of imaginary part of electric modulus (M”) as a function of temperature and frequency are plotted in Fig. S6. Two distinct peaks at high temperature, low frequency, and low temperature, high frequency come from the interfacial polarization (Maxwell–Wagner–Sillars effect, which is normally termed as MWS polarization) and amorphous molecular relaxation, respectively [25, 49, 50]. Clearly, the peak of molecular relaxation is barely affected by BNNS layer. Of particular importance is that the peak of MWS polarization of PVDF is distinctly suppressed in PBPBP films.

To better visualize this vibration, M” as s function of frequency under different temperature of five samples derived from Fig. S6 is plotted in Fig. S7. Apparently, the peak of M” shifts to higher frequency with temperature goes up, which is attributed to hopping process of ions at high temperature [51]. In addition, the activation energy (*E*_a_) of MWS polarization can be calculated by using Arrhenius equation, $\mathrm{ln}{f}_{max}=\mathrm{ln}{f}_{0}-\frac{{E}_{a}}{kT}$, where *f*_max_ is the peak frequency of M”, *f*_0_ is the pre-exponential factor, *T* is absolute temperature, and *k* is the Boltzmann constant [47, 52]. As shown in Fig. 3d, the *E*_a_ of PBPBP films (from 1.26 to 1.48 eV) are much higher than pristine PVDF (0.99 eV), indicating that more energy is required for the occurrence of MWS polarization in PBPBP films. In other word, comparing with PVDF, the space charge in PBPBP films is harder to migrate to the interfacial regions, leading to suppressed dielectric loss.

### Energy Storage Performance of PBPBP Films

**Fig. 4:**
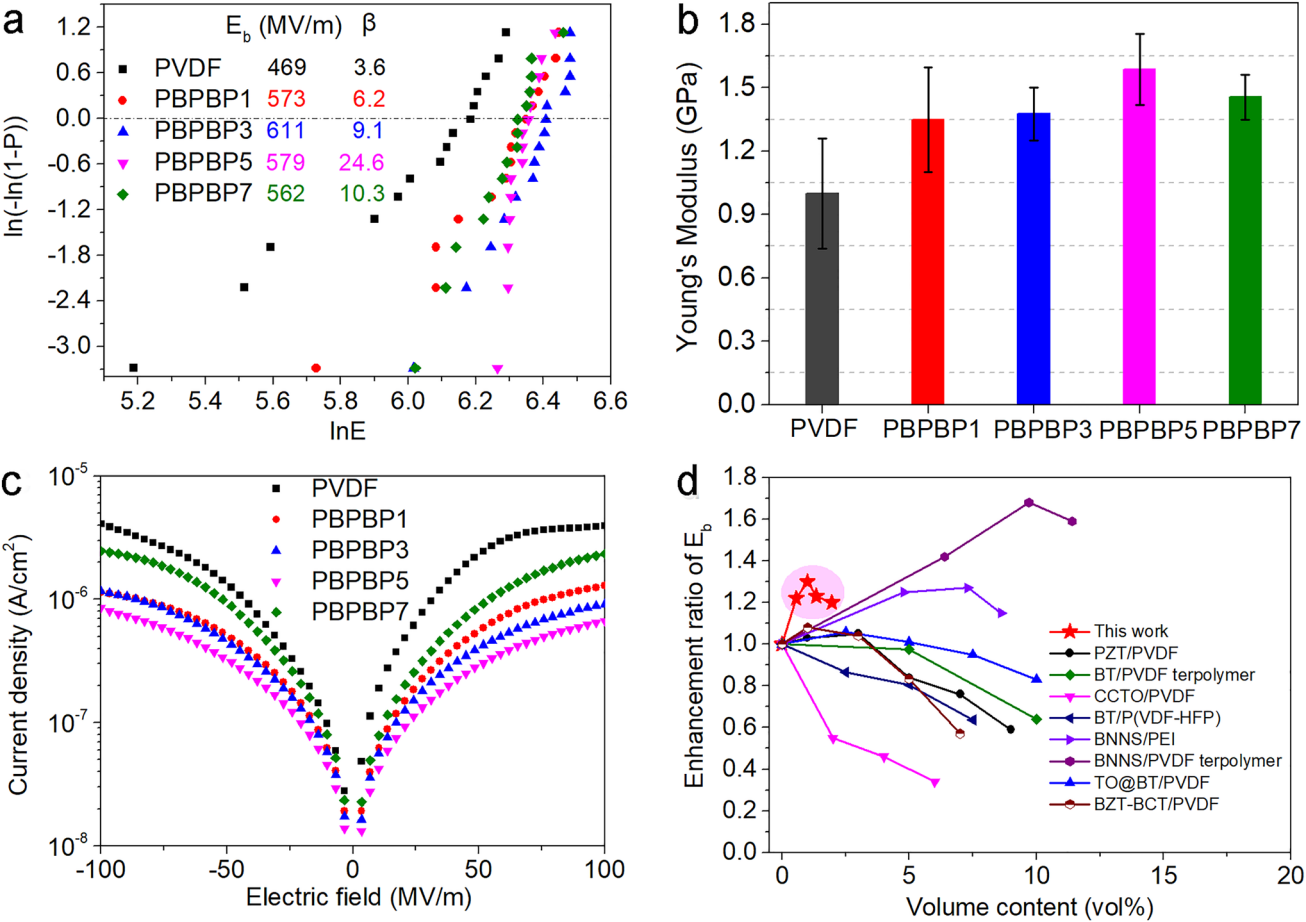
**a** Weibull statistical distribution plots, **b** Young’s modulus, and **c** I-V curves of PVDF and PBPBP films. **d** Comparison of enhancement ratio of *E*_b_ (compared to polymer matrix) of this work with previous reported work [19, 22, 55–60]

The breakdown strength of dielectric samples are evaluated by two-parameter Weibull statistical distribution equation, $P\left(E\right)=1-exp[-{\left(\frac{E}{{E}_{b}}\right)}^{\beta }]$ (where *P(E)* is cumulative probability of electrical breakdown, *E* denotes the experimental result of every plot, *E*_*b*_ is the breakdown strength, representing the probability of dielectrics breakdown under this electric field is 63.2%, *β* is shape parameter which reveals reliability of the data) and results are shown in Fig. 4a. Clearly, pristine PVDF possesses low *E*_*b*_ (469 MV m^−1^) and low *β* (3.6). All PBPBP films exhibit notably improved breakdown strength and higher shape parameter than PVDF, in which PBPBP3 exhibits the highest *E*_*b*_ of 611 MV m^−1^ and PBPBP5 possesses the highest *β* of 24.6, respectively. The significantly increased breakdown strength can be ascribed to two aspects. First is the Young’s modulus of five films (derived from stress–strain curves in Fig. S8). According to the electrochemical breakdown theory, the breakdown strength of dielectrics is proportional to its Young’s modulus [53, 54]. As shown in Fig. 4b, comparing with pristine PVDF (1.2 GPa), the Young’s modulus of PBPBP films exhibits a gradual increase and reaches 1.6 GPa for PBPBP5. Therefore, the improved Young’s modulus of PBPBP films leads to greater ability to block mechanical deformation under applied electric field, thus accounting for their substantially improved breakdown strength. Furthermore, the advanced breakdown strength also derives from the suppressed leakage current shown in Fig. 4c. The current densities of PBPBP films exhibit a distinct decrease comparing to PVDF. For instance, the leakage current density of the leakage current density of PBPBP5 is 6.65 × 10^–7^ A cm^−2^, which is nearly one order of magnitude lower than that of PVDF (3.92 × 10^–6^ A cm^−2^).

In addition, thus greatly elevated breakdown strength is accomplished at fairly low filler content (i.e., 0.56, 1.00, 1.35, 1.96 vol%). This is advantageous to dielectric polymer composites because less inorganic filler means less polymer/filler interface, which may leads to fewer defects and reduced negative effect on the mechanical property of polymer hosts. Therefore, to reveal its significant efficiency in lifting breakdown strength, the enhancement ratio of *E*_*b*_ of this work is compared with previous reported results [55–62], as shown in Fig. 4d. It can be seen that the volume fraction of previous reported work varied from 1 to over 12%. On one hand, the enhancement ratio of *E*_*b*_ hardly succeeds 1.15 even at the optimal dopant of high dielectric constant nanofiller. On the other hand, the enhancement ratio of *E*_b_ reaches 1.27 and 1.68 when nanocomposites are filled with 7.3 and 9.7 vol% BNNS, separately. Nevertheless, exfoliation of high quality BNNS is still time-consuming and tedious [63], such high dopant is hardly applicable in mass production considering high dopant will also cause poor processibility. For comparison, in this work, when the BNNSs content is merely 0.56 vol%, the enhancement ratio of *E*_b_ is higher than 1.2 and reaches 1.3 when the BNNSs content volume is 1%, suggesting that two BNNS layers have remarkable efficiency in improving the breakdown strength of nanocomposite over random dispersed BNNSs.

**Fig. 5:**
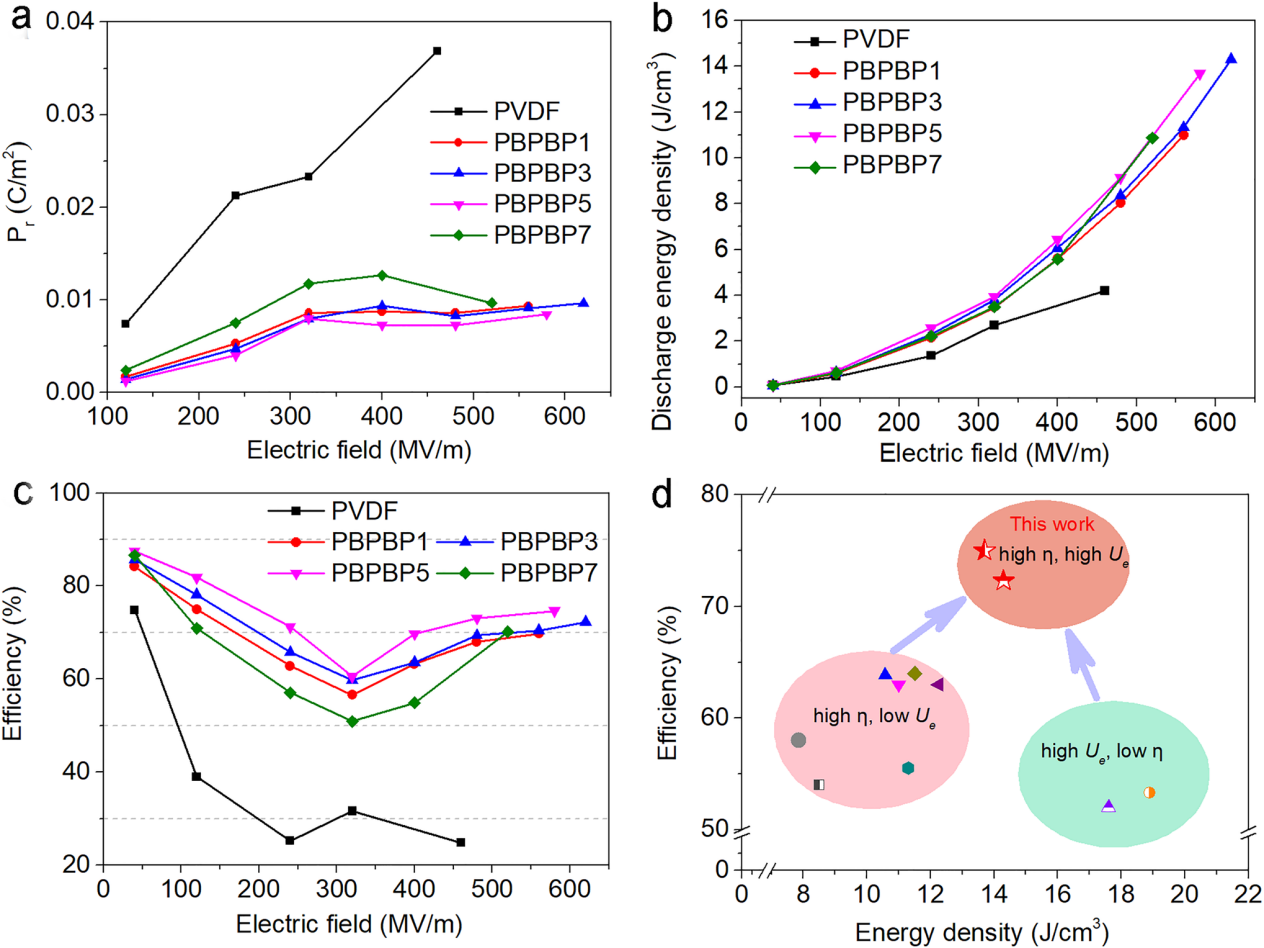
**a** The remnant polarization (*P*_r_) as a function of electric field, **b** discharged energy densities and **c** charge–discharge efficiencies as a function of electric field of PVDF and PBPBP films. **d** Comparison of efficiency and maximum discharged energy density of PBPBP3 and PBPBP5 with previous reported results [57, 60, 64–70]

Afterwards, the energy storage capabilities of as-prepared five films are evaluated by the D-E (electric displacement-electric field) loops shown in Fig. S9. One can see that all PBPBP films exhibit much slender D-E loops than pristine PVDF, which is mainly ascribed to the significantly restrained remnant polarization (*P*_*r*_) shown in Fig. 5a. For example, the *P*_*r*_ of PVDF rapidly grows over 0.03 C m^−2^ as applied electric field exceeds 400 MV m^−1^. Meanwhile, for PBPBP3, its *P*_*r*_ remains an extremely low value of 0.0096 C m^−2^ under an electric field as high as 620 MV m^−1^. Since the dielectric loss under high electric field mainly origins from conduction loss [71, 72], this indicates that two BNNS layers served as effective electron blocker, significantly inhibited the conduction loss. In addition, due to the much improved breakdown strength, compared to pristine PVDF, PBPBP films can bear higher electric field.

The discharged energy densities (*U*_*e*_) and charge–discharge efficiencies (*η*) of as prepared five samples are calculated based on the original data of D-E loops and plotted in Fig. 5b, c. Clearly, the *η* of PVDF drops to lower than 40% at 120 MV m^−1^ and then decreases to 30% and even 20% as increasing the electric field. Benefiting from the suppressed *P*_*r*_, the *η* of PBPBP films exhibit a distinct lift to over 70% even the electric field is as high as 600 MV m^−1^. In addition, the *η* of PBPBP films first exhibits a gradual decrease to nearly 50% and then goes up, which is consistent with the trend of *P*_*r*_ shown in Fig. 5a. Brought by significantly boosted *E*_*b*_ and much promoted *η*, the *U*_*e*_ of PBPBP films show an extraordinary improvement compared to pristine PVDF, as shown in Fig. 5b. For instance, PBPBP3 has the highest *U*_*e*_ of 14.3 J cm^−3^, which is 3.4 times of PVDF (4.2 J cm^−3^).

To further clarify the significance of this work, we compared PBPBP3 and PBPBP5 with previous reported PVDF based nanocomposites by the most important two parameters of dielectric energy storage materials, *U*_*e*_ and *η* [57, 60, 64–70]. As shown in Fig. 5d, previous reported works possess a *U*_*e*_ from 7.8 to 18.9 J cm^−3^ with none *η* exceeds 65%. However, the *U*_*e*_ and *η* of PBPBP3 and PBPBP5 are 14.3 J cm^−3^ and 72%, 13.7 J cm^−3^ and 75%, respectively. Such a high improved *η* is important because high efficiency means less energy loss, and hence long lifetime for dielectric capacitors. In addition, thus superior *U*_*e*_ and *η* are achieved at low BNNSs content of 1 and 1.35 vol%, indicating the remarkable efficiency of BNNS layer in boosting energy storage performance of dielectric nanocomposites.

### Mechanism of Improved Energy Storage Performance Brought by BNNS Layer

To underlying the mechanism of BNNS layers on the improved energy storage of dielectric composites, first-principles calculations are performed to evaluate the energy band structure of BN and PVDF, which is shown in Table S2. The electron affinity is regarded as the energy difference between the vacuum level and conduction band minimum or LUMO level [36]. Therefore, the electron affinity of BN and PVDF are 1.18 and − 5.224 eV, respectively, thus triggering the electron traps at the interface between BN and PVDF, which has been confirmed in experimental results shown in Fig. 1o.

**Fig. 6:**
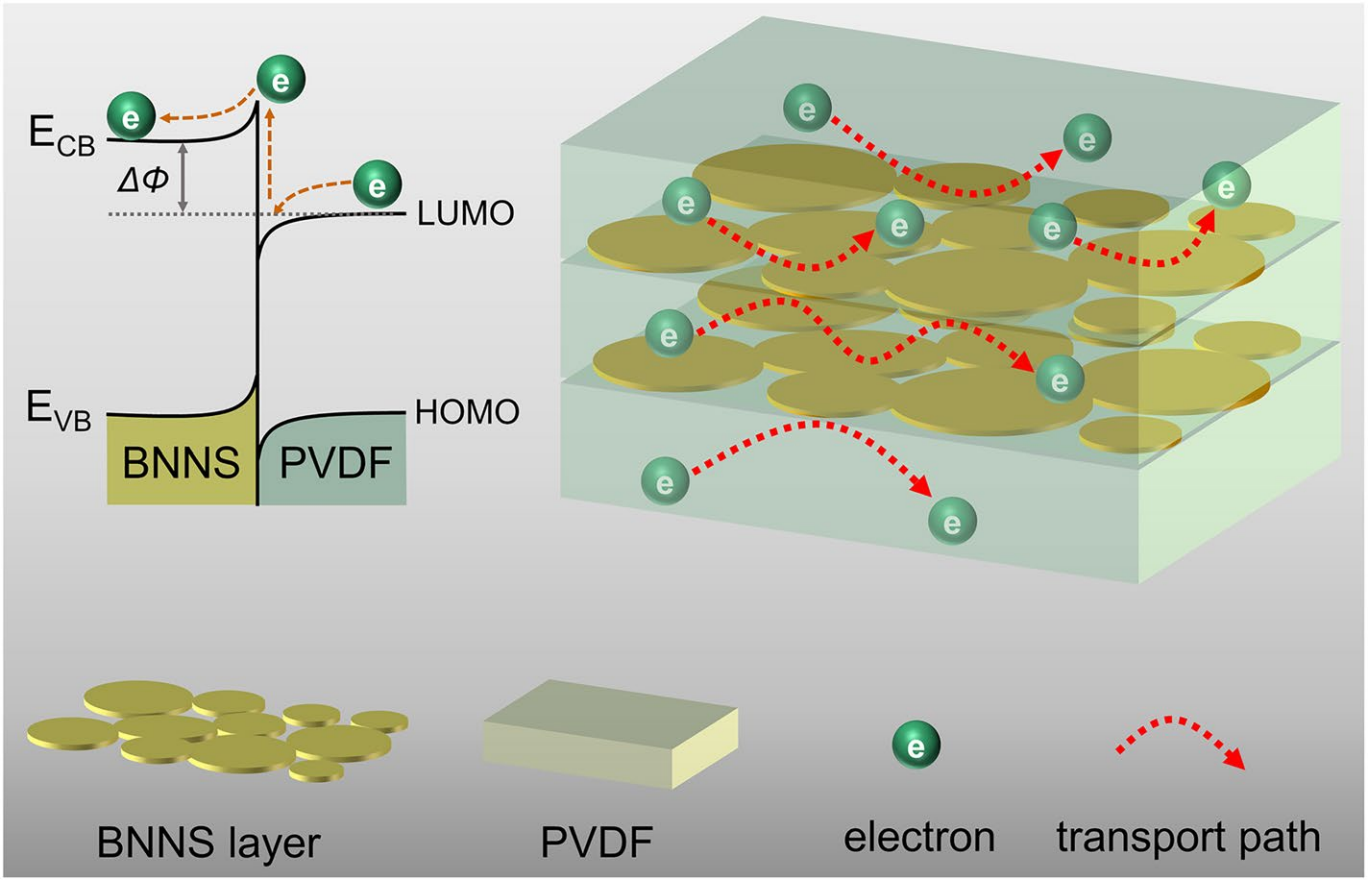
Schematic illustration of restricted electron transportation in multilayered nanocomposite

Furthermore, the Fermi level of BN is higher than that of PVDF (Fig. S10a). After contact, the electrons in BN will transfer into PVDF [73], leading to equivalent Fermi energy level shown in Fig. S10b. Nevertheless, the conduction band minimum of BNNS is still higher than LUMO of PVDF, which creates an energy barrier (*ΔΦ*) preventing the electron transfer from PVDF to BN. As depicted in Fig. 6, electrons in LUMO of PVDF (mainly comes from electrode injection) need to climb over *ΔΦ* and then migrate to the conduction band minimum of BNNS. Considering BNNSs are assembled into compact layer between PVDF layers, one can speculate that this energy barrier has been maximized to prevent electrons in PVDF from penetrating through BNNS layers (as shown in Fig. 6), leading to significantly suppressed leakage current density of multilayered nanocomposites. Along with the improved Young’s modulus, BNNS layers lead to substantially advanced energy storage performance of multilayered nanocomposites.

## Conclusions

In summary, high conduction band inorganic layers for substantially improved electrical energy storage performance of PVDF nanocomposite is demonstrated in this work. A series of multilayered nanocomposites with alternately arranged BNNS and PVDF layers are manufactured by a facile solution-casting strategy. Results show that BNNS layers generate deep traps, significantly suppress the leakage current and restrain the remnant polarization of nanocomposite. Energy level and band simulation results reveal that an energy barrier locates at the interface between BN and PVDF, preventing electrons in PVDF from migrating through BNNS layer, which is consistent with experimental results. In addition, BNNS layers increase the Young’s modulus of multilayered nanocomposites. As a result, breakdown strength and discharged energy density of multilayered nanocomposites are remarkably improved. For instance, the multilayered nanocomposite possesses an outstanding breakdown strength of 611 MV m^−1^ and an excellent discharged energy density of 14.3 J cm^−3^, which are 119 and 177% of the BNNSs randomly dispersed nanocomposite (515 MV m^−1^, and 8.1 J cm^−3^). Since such an excellent energy storage performance promotion is accomplished with only 1 vol% BNNSs, the current work offers a new paradigm for design and manufacture of high energy density flexible dielectric films in the near future.

## Supplementary Information

Below is the link to the electronic supplementary material.

Supplementary file1 (PDF 1516 kb)
