# Supplementary material for: High Conduction Band Inorganic Layers for Distinct Enhancement of Electrical Energy Storage in Polymer Nanocomposites
Source: Nanomicro Lett. 2022 Jul 25;14:151. doi: 10.1007/s40820-022-00902-9 (PMC9314523; doi:10.1007/s40820-022-00902-9)
Supplement: Supplementary file 1 — Supplementary file1 (PDF 1516 kb) [file 40820_2022_902_MOESM1_ESM.pdf]

Supporting Information for

# High Conduction Band Inorganic Layers for Distinct Enhancement of Electrical Energy Storage in Polymer Nanocomposites

Yingke Zhu[1], Zhonghui Shen[2], Yong Li[3], Bin Chai[1], Jie Chen[1], Pingkai Jiang[1], Xingyi Huang[1, *]

[1]Department of Polymer Science and Engineering, Shanghai Key Laboratory of Electrical Insulation and Thermal Ageing, State Key Laboratory of Metal Matrix Composites, Shanghai Jiao Tong University, Shanghai 200240, People's Republic of China

[2]State Key Laboratory of Advanced Technology for Materials Synthesis and Processing, Center of Smart Materials and Devices, Wuhan University of Technology, Wuhan 430070, People's Republic of China

[3]Institute of Applied and Physical Chemistry and Center for Environmental Research and Sustainable Technology, University of Bremen, Bremen 28359, Germany

*Corresponding author. E-mail: xyhuang@sjtu.edu.cn (Xingyi Huang)

## Supplementary Tables and Figures

**Table S1** The values of parameters used in this simulation at 298 K and 300 MV $m^{-1}$

| Parameters | Values in matrix | Values in filler |
|---|---|---|
| Schottky barrier (eV) | 1.0 | / |
| Trap Depth $\xi$ (eV) | 0.12 (at interfaces) | / |
| Electron mobility ($cm^2$/V·s) | $9\times10^{-11}$ | 10 (in plane) 0.01 (out of plane) |
| Dielectric constant | 16 | 5 |

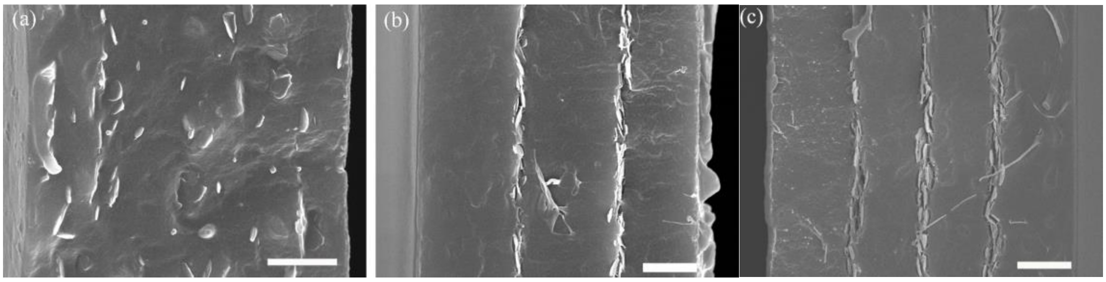


**Fig. S1** SEM images of **a** BNNS random dispersed composite, **b** composite with two aligned BNNS layers, **c** composite with three aligned BNNS layers(all scalr bars are 2 μm)

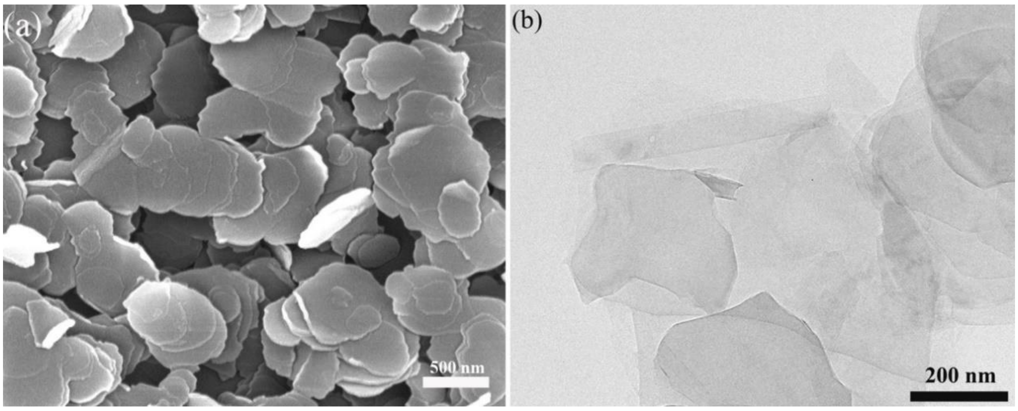


**Fig. S2 a** SEM and **b** TEM image of BNNSs

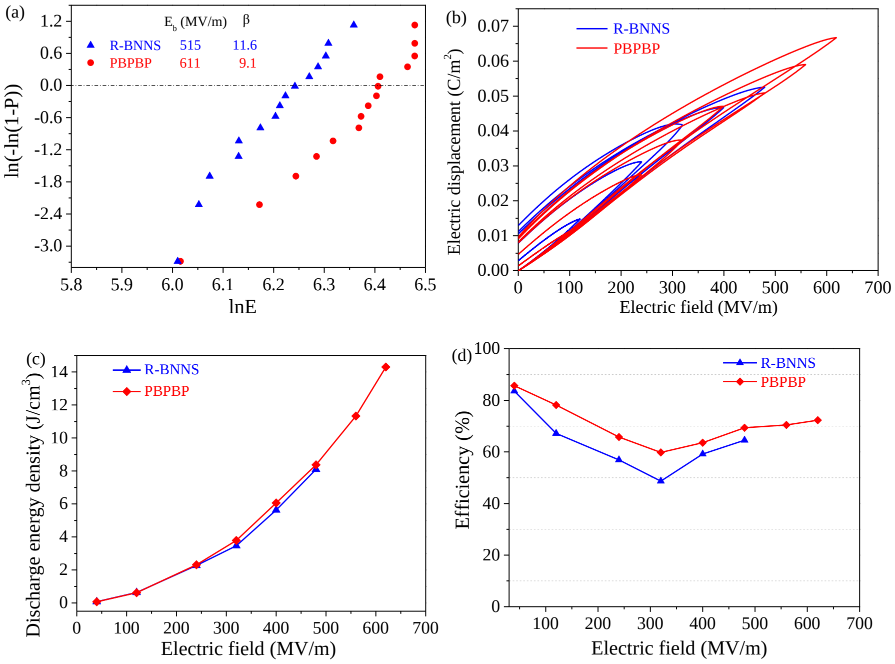


**Fig. S3** Comparison of **a** Weibull breakdown strength, **b** D-E loops, **c** discharged energy density, and **d** charge-discharge efficiency of R-BNNS and PBPBP

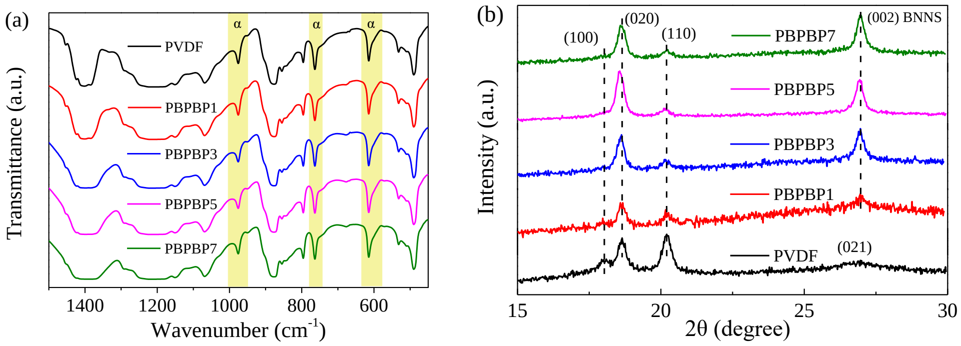

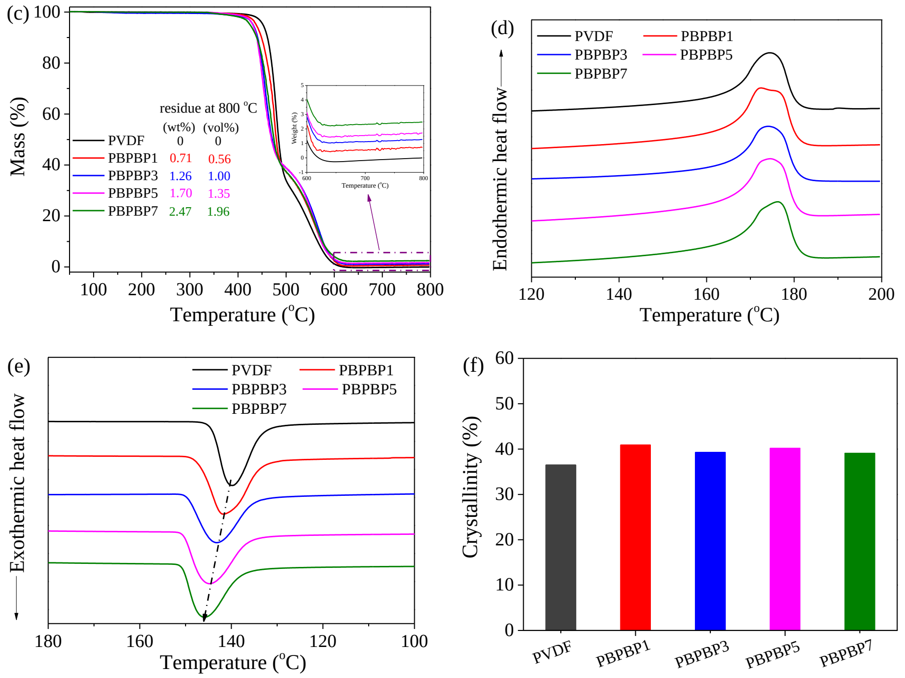


**Fig. S4 a** FT-IR, **b** XRD, **c** TG, **d** DSC heating, **e** DSC cooling, and **f** crystallinity spectra of PVDF, and PBPBP films

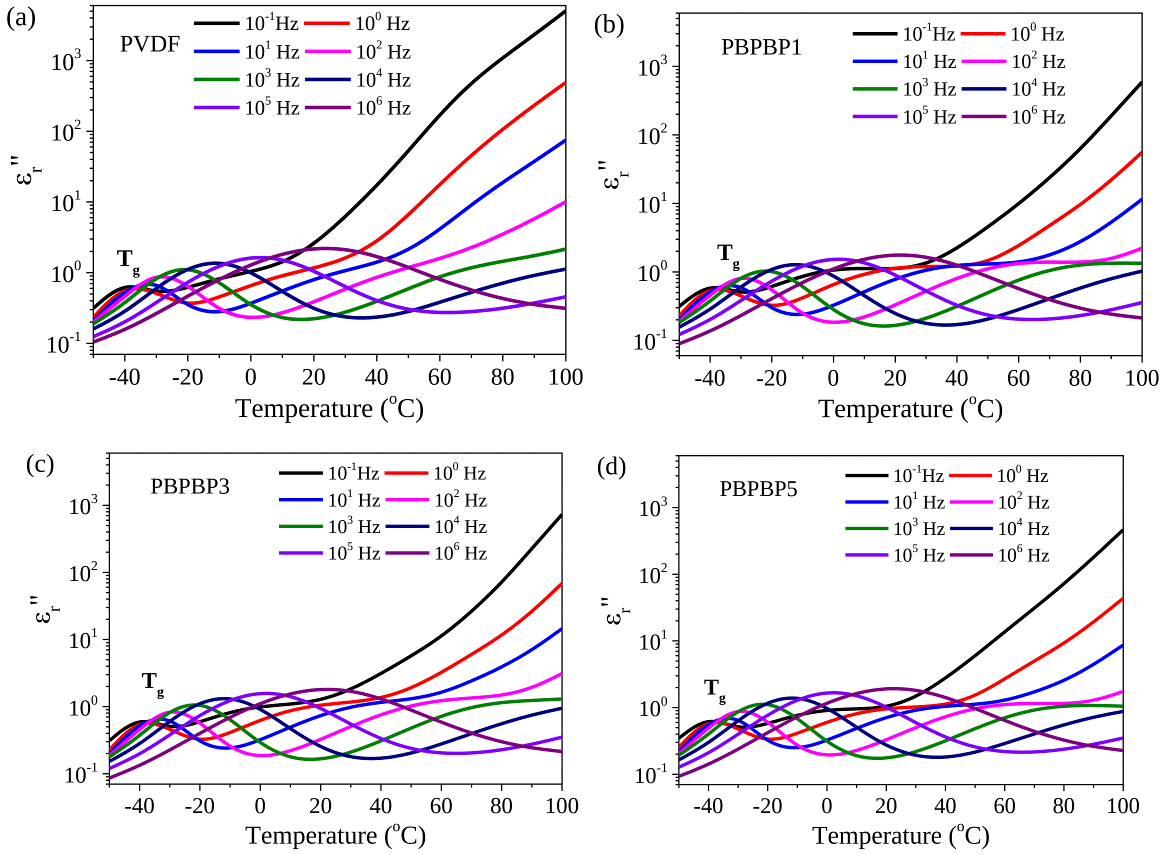

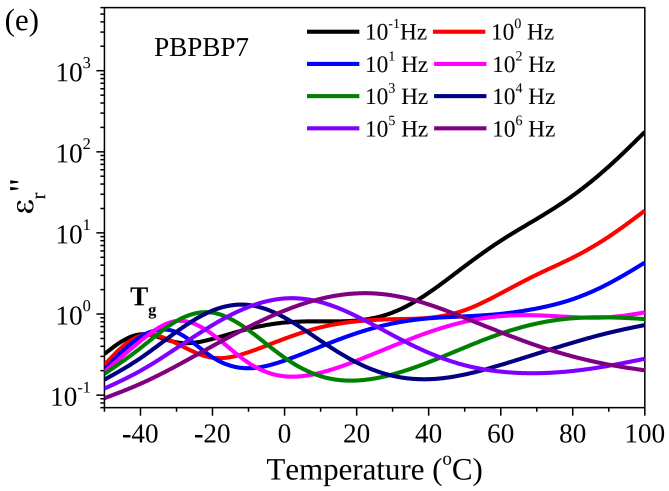


**Fig. S5** Imaginary part of dielectric constant ($\varepsilon_r''$) of **a** PVDF, **b** PBPBP1, **c** PBPBP3, **d** PBPBP5, **e** PBPBP7 under various temperature

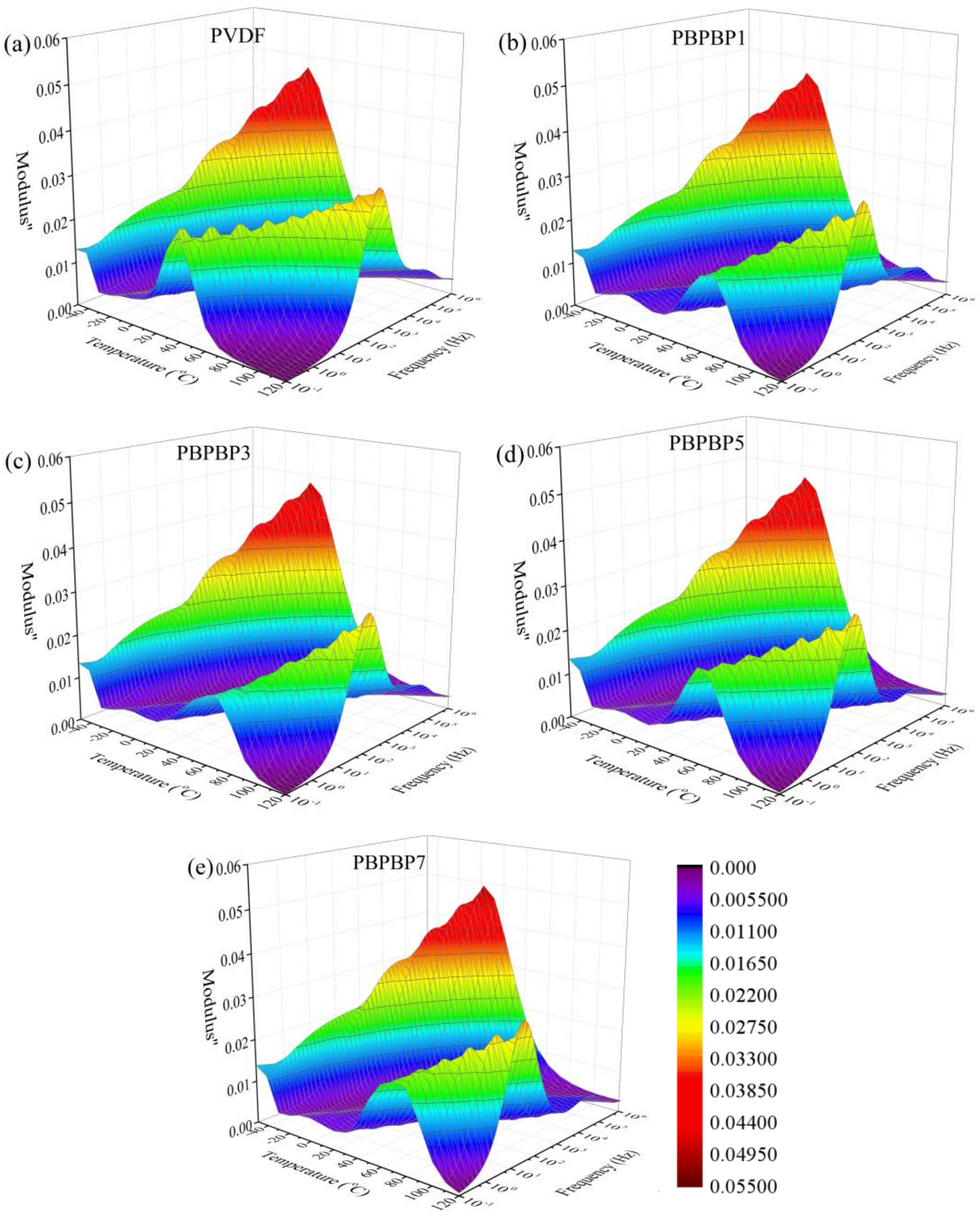


**Fig. S6** Frequency dependance of the imaginary part of electric modulus (M") of PVDF and PBPBP films under various temperature

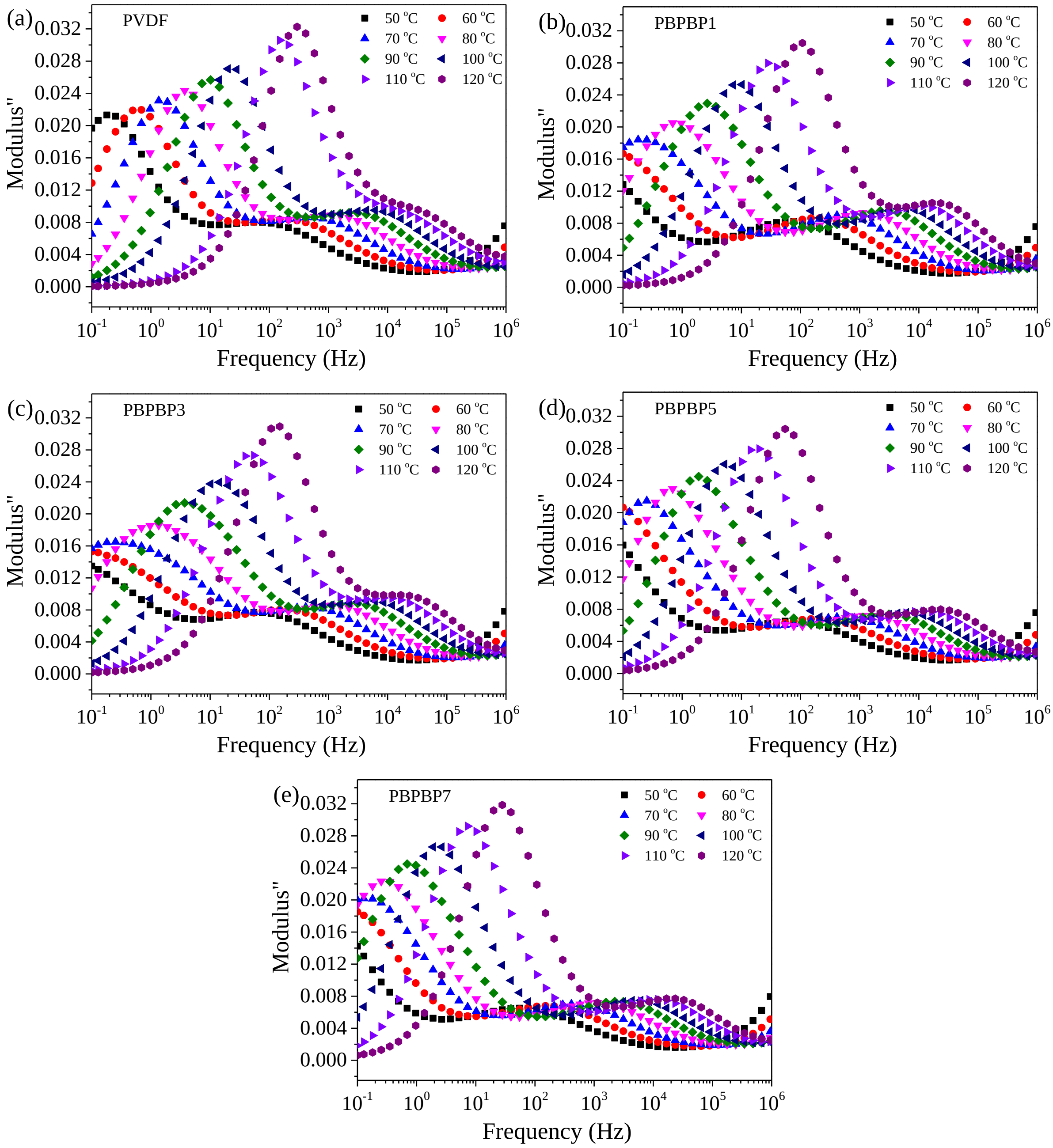


**Fig. S7** The imaginary part of electric modulus (M") as a function of frequency under various temperature of **a** PVDF, **b** PBPBP1, **c** PBPBP3, **d** PBPBP5, and **e** PBPBP7, respectively

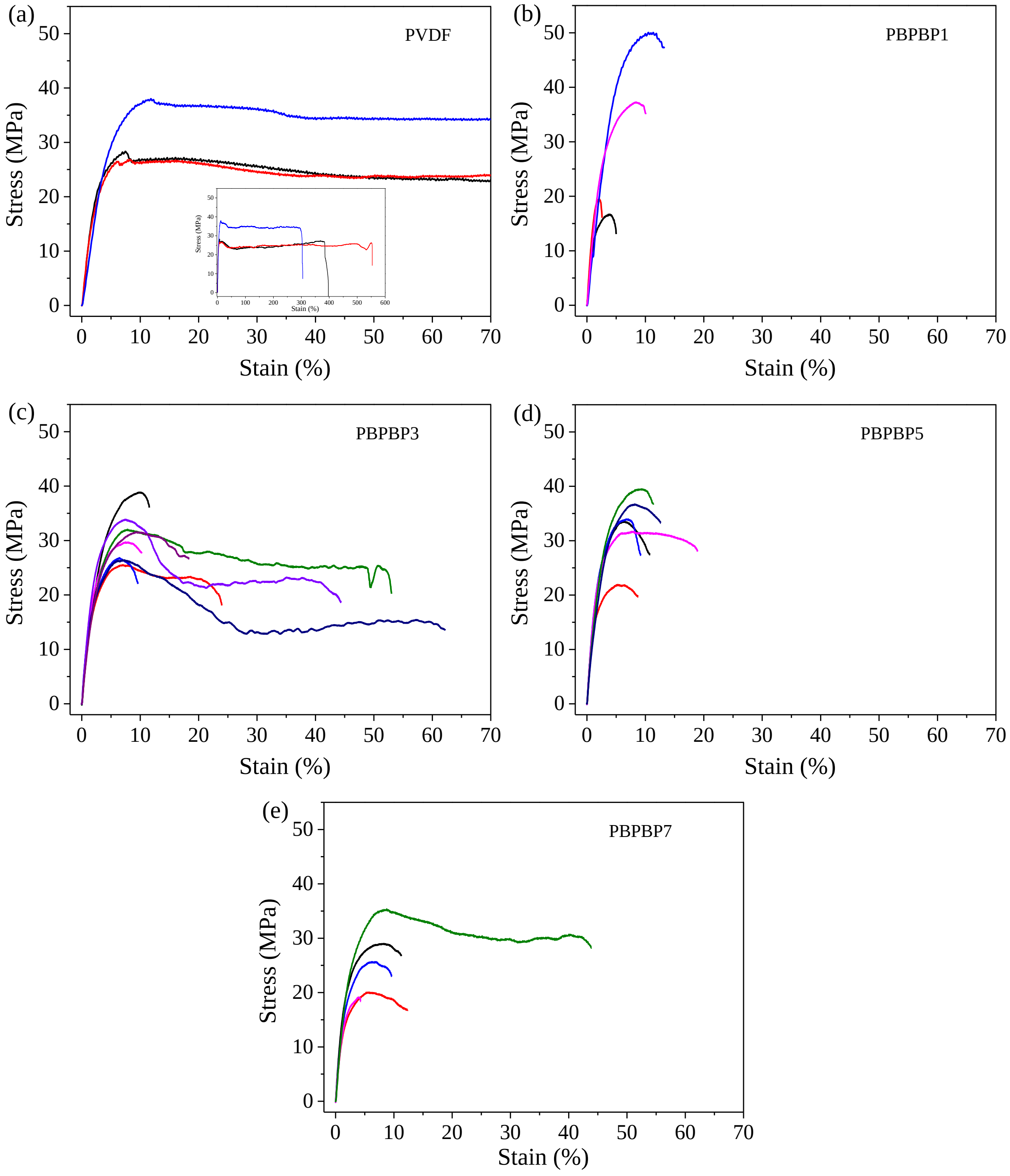


**Fig. S8** The stress-strain curves of PVDF and PBPBP films

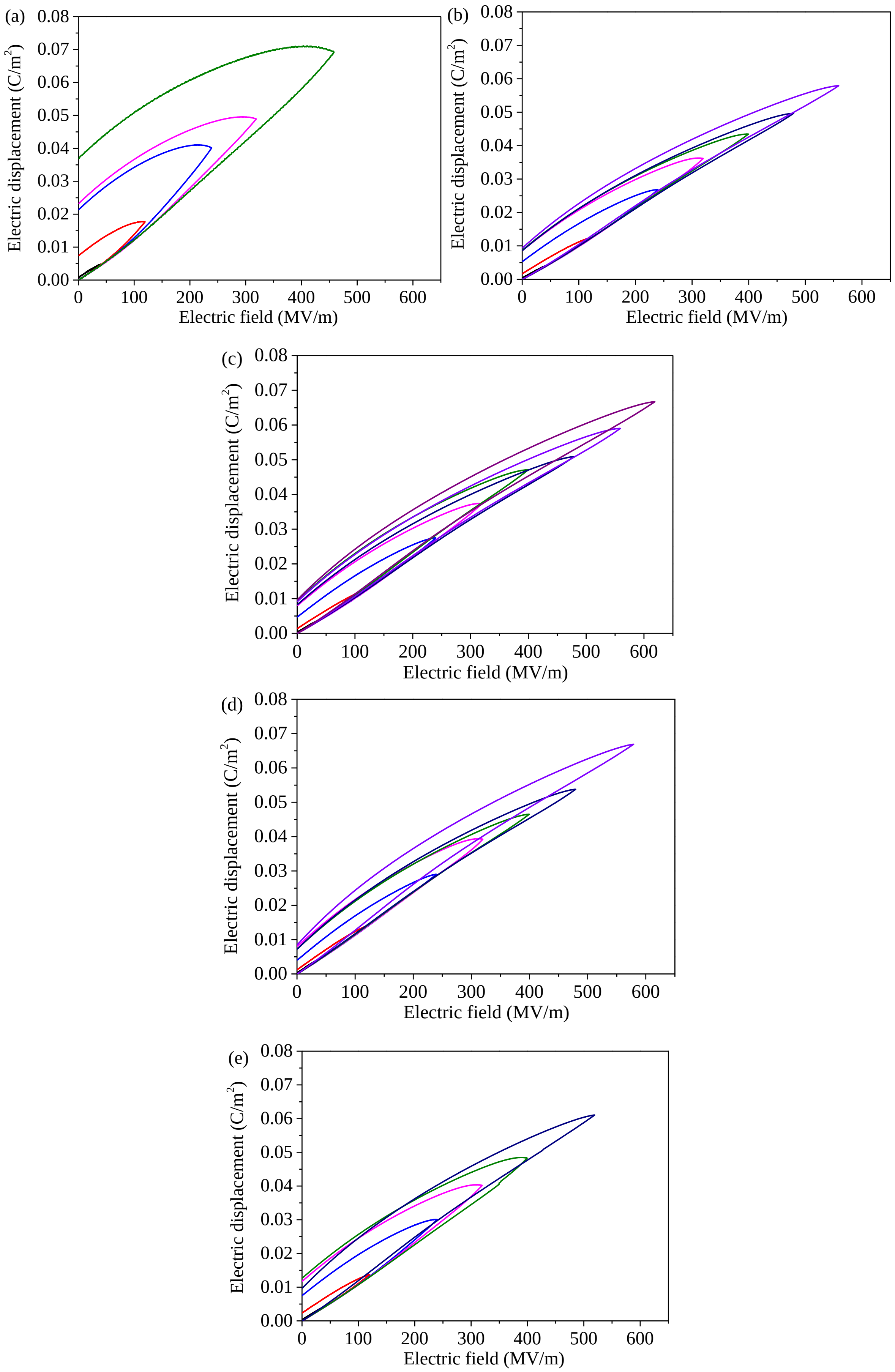


**Fig. S9** D-E loops of **a** PVDF, **b** PBPBP1, **c** PBPBP3, **d** PBPBP5, and **e** PBPBP7, respectively

**Table S2** Electronic property parameters of BNNS and PVDF calculated by the first-principles calculations

| Materials | Electronic property parameters | | |
|---|---|---|---|
| BNNS | Vacuum level | $E_{vac}$ | 5.961 |
| | Conduction band | $E_{CB}$ | 7.141 |
| | Valence band | $E_{VB}$ | 2.825 |
| | Work function | φ | 5.540 |
| | Fermi level | $E_F$ | 3.040 |
| PVDF | Vacuum level | $E_{vac}$ | 5.200 |
| | LUMO level | LUMO | -0.224 |
| | HOMO level | HOMO | -6.852 |
| | Work function | φ | 7.861 |
| | Fermi level | $E_F$ | -6.602 |

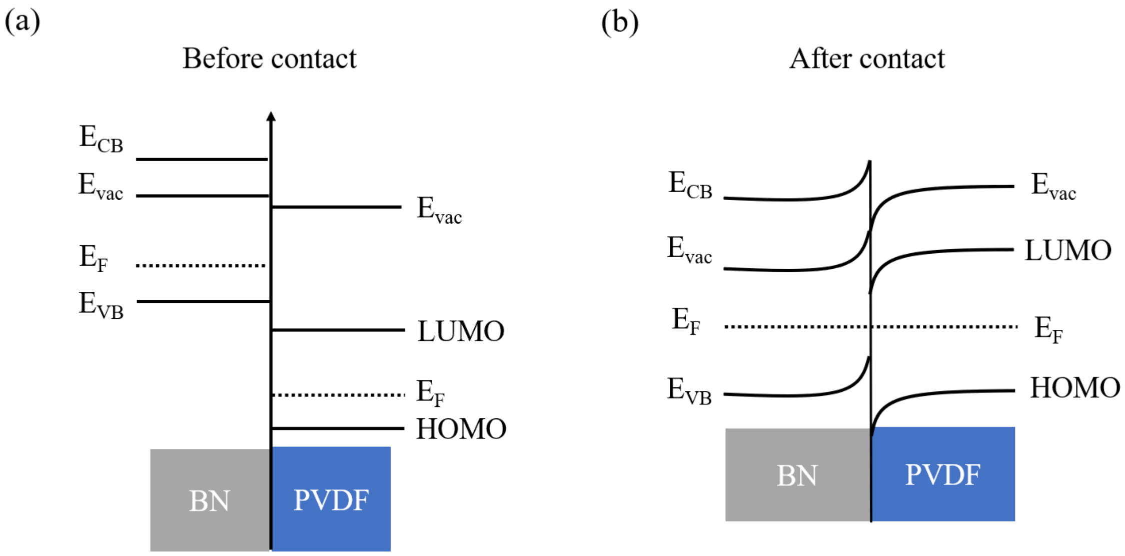


**Fig. S10** Energy band strcuture of BN and PVDF, (**a**) before contact and (**b**) after contact

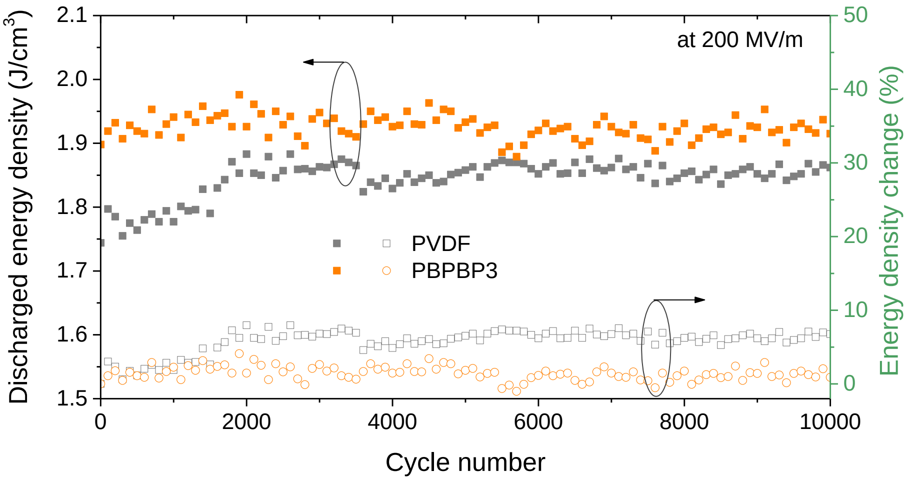


**Fig. S11** Cyclic charge-discharge stabilities of PVDF and PBPBP3 under 200 MV $m^{-1}$
